# New Potentiometric Screen-printed Sensors for Determination of Trimebutine Drug in Tablets, Serum and Urine Samples

**DOI:** 10.22037/ijpr.2019.13892.11970

**Published:** 2020

**Authors:** Tamer Awad Ali, Gehad Genidy Mohamed, Adel Zaki El-Sonbati, Mostafa Amin Diab, Ahmed Mohmed Elkfass

**Affiliations:** a *Egyptian Petroleum Research Institute (EPRI), 11727, Cairo, Egypt. *; b *Department of Chemistry, Faculty of Science, Cairo Un4ersity, 12613, Giza, Egypt. *; c *Department of Chemistry, Faculty of Science, Damietta Un4ersity, Damietta, Egypt.*

**Keywords:** Modified screen-printed electrode, Determination of trimbutine maleate, Pharmaceutical preparations, Potassium tetrakis(*p*-chlorophenyl) borate ionophore, Biological fluids

## Abstract

A new sensit4e and select4e modified screen printed electrodes (MSPEs) and carbon paste electrodes (MCPEs) were studied in order to determine trimbutine maleate (TM) in pure, tablets, urine, and serum samples. These sensors were embodied with multiwalled carbon nanotubes (MWCNTs) since it improved the quality of the sensors in presence of potassium tetrakis (*p*-chlorophenyl) borate (KTpClPB) ionophore. A good Nernstian response for the constructed sensors, at optimum paste composition, was exhibited for determination of TM in concentration range of 1.5 × 10^-7^ - 1.0 × 10^-2 ^and 1.0 × 10^-7^- 1.0 × 10^-2^ mol L^-1^ at 25 °C with detection limit of 1.5 × 10^-7 ^and 1.0 × 10^-7^ mol L^-1^ for MCPE and MSPE, respect4ely. It seemed that the potential of the electrodes was independent on *p*H in the range of 2.0-8.0, 2.0-8.5, 2.0-8.5, and 2.0-9.0 g4ing slope as 56.77 ± 1.11, 57.82 ± 0.54, 57.95 ± 0.37, and 58.99 ± 0.28 mV decade^-1^ for electrodes 1, 2, 3 and 4, respect4ely. MCPEs and MSPEs gave response time about 8 and 6 s with long lifetime (more than 3 and 5 months), respect4ely. A high select4ity of sensors was observed for TM regarding to a large number of interfering species. The constructed sensors were successfully applied for determination of TM in pure form, its pharmaceutical preparations and biological fluids using standard addition, calibration, and potentiometric titration methods with high precision and accuracy. The results showed a good agreement between the proposed method and the HPLC official method.

## Introduction

**Figure 1:**
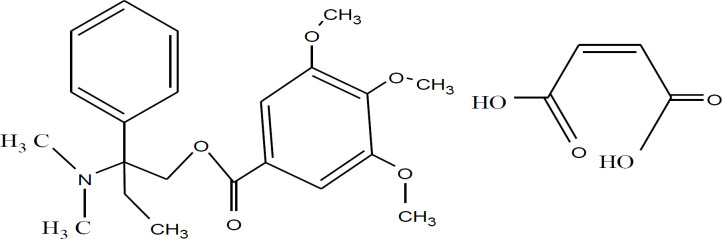
Chemical structure of trimebutine maleate (TM).

Trimbutine maleate (TM) is an antispasmodic drug and can be named as 2-dimethyl-amino-2-phenylbutyl-3,4,5-trimethoxy-benzoate hydrogen maleate in IUPAC, Figure 1. It can be used in treating of irritable bowel syndrome, hyperkinetic and hypokinetic motility disorders and various gastrointestinal (GI) disorders including postoperative ileus (1-3). It has antispasmodic actions on the GI. It has an unusual regulation with dual effects on GI function (3). It is the widely known prokinetic agent which has been established to effect on the smooth muscle of the GI tract (4). TM displayed a weak opioid properties (5) and affects the activity of visceral afferent nerves in the rat (6). TM may frustrate spontaneous contractions depending on the previous constrictive activity during making (7). TM could be active against rectal hyperalgesia induced by local inflammation and stress in rats (8). Due to its weakness against ability to active the intestinal opioid receptor TM shows antispasmodic activity. TM drug shows Local narcotic activity and this may be due to its blocking of sodium channels (9). Improvement in the pharmacological effects of TM could be improved and developed by forming inclusion complex between TM and *p*-sulfonatothiacalix(6)arenes (SCX6) (10).

Drugs need to be high sensitive determined with high sensitive methods than the reported methods. Many prior studies developed several analytical methods for the determination of TM and its metabolites in biological fluids. These methods included high-performance liquid chromatography using UV, mass spectrometry, spectrofluorimetry, spectrophotometry, flow injection, and fluorescence detection (11-18). Only one paper represented the analysis of TM and its metabolites in human plasma using GC–MS (19). TM could be also quantitatively determined in plasma using capillary zone electrophoresis methods for chiral separation and in capsules, rat plasma and tissues (20-22). On the other way, there are some ways found to determine TM in dosage forms such as spectrophotometric methods that used to determine TM by forming ion-pair with bismuth(III)-iodide, bromocresol green, bromophenol blue and bromothymol blue; and by charge-transfer complexation with iodine. Also, high performance liquid chromatographic (HPLC) methods were hired for the assay of TM and its metabolite in human plasma and related impurities (20, 23-25). It can be easily degraded as it is considered as an ester type antispasmodic; nevertheless, TM could not be determined in the presence of its degradation products. Its division in tissue of dogs, the rats and mice could be studied using radiometric analysis with either ^14^C or ^3^H labeled TM with different division patterns of radioactivity between the labeled compounds due to the metabolism of the drug (13). Nevertheless, these methods were not enough to study pharmacokinetic of TM and its metabolites due to some failures or limitations such as laborious, boring and not cheap, require warming or extraction and many facilities, rare sensitivity, consuming much time and small range of determination. 

Ion-selective electrodes (ISE) were used to determine selectivity and activity of a given ion irrespective of other ions found in the solution (26-29). Clearly, ISE However, these methods suffer from several disadvantages such as time-consuming, required expensive instrumentation and sample preparation, required large infrastructure back up and they involved a large number of complicated pretreatment steps for analysis (30-32). Electrochemical techniques have proved to be a powerful and versatile method for the sensitive determination of biologically important drug compounds and related molecules in pharmaceutical dosage forms and human body ﬂuids. Owing to their extreme simplicity, low cost, relatively short analysis time and high sensitivity and speciﬁcity as compared to other techniques, electroanalytical techniques are of great importance in the ﬁeld of drug analysis (32). Selective analytical methodologies, which are easily operated and involve harmless reagents, cost effective equipment, have therefore been proposed as alternative to standard methods. Potentiometric sensors based on ion-selective electrodes are especially suited for such determination because they offer advantages such as selectivity, sensitivity, good precision, simplicity, and low cost (21). Ion-selective electrodes (ISEs) are classified as electrochemical transducers that respond continuously, selectively and directly to the activity of the analyte in solution which have some advantages. They can be used without previous extraction of samples, easily fabricated, low cost, and fair accuracy (33). They are the most suitable method for direct determination and they can be used as indicator sensors in titrations if compared with other analytical techniques. Also there is no effect on the studied solutions using these membrane electrodes (34-41). One of the simplest method of ISEs is potentiometric detection which has many advantages such as instrument simplicity and can be used for online analysis with high velocity. The electrodes can be easily prepared with large concentration range, fast response time, high selectivity and cheap (42-47).

Carbon sensors have many types such as glassy carbon, carbon paste, carbon films, screen-printed carbon and carbon fiber and can be largely used in analytical electrochemistry as they have many benefits such as cheap, relatively inert electrochemistry, wide potential window, and electrocatalytic activity for a range of redox reactions. 

A type of ion-selective electrodes called carbon paste electrodes (CPEs) that is largely used for the electrochemical designations of a different biological and pharmaceutical species because of their advantages and benefits (48-50). CPEs were discovered in 1958 by Ralph N. Adams and have earned much consideration in electrochemical field (51-58). Such sensor was composed of graphite including high viscosity organic liquid as the pasting liquid. One of the most preferable candidates , used for the preparation of electrocatalytic nanocomposite materials is called multi wall carbon nanotubes (MWCNTs) [59, 60]. MWCNTs can be ascribed to heir nanometer dimensions, the electronic structure, and the topological defects found in the surfaces of MWCNTs. One of the most advantages of MWCNTs is increasing of the effective area of the sensor (56, 57 and 59). So, their combination into electrochemical sensors gave many advantages such as resistance to surface fouling, reduction of over-potentials, low detection limits, and high sensitivities (45, 46). 

Another type of ion-selective electrodes called screen-printed electrodes (SPEs) which is developed and used in production of electrochemical sensors/ biosensors (61) depending on screen-printing technology. Different types of inks on different ceramic or plastic substrates can be printed producing SPEs. They have many advantages as widely accessible, simplicity and cheapness disposable electrochemical sensors (51, 55 and 62). Also they were used for miniaturization of electrochemical analytical systems. These sensors can be easily developed and modified in different ways to be more suitable for measuring multiple biological samples, as they required a small sample volume.

Potentiometric determination using screen-printed sensors were selected to determine drugs but there were main challenges for the electroanalysis, and of drug residuals in biological fluids, sample cleanup, and pretreatment as well as sample consumption (63, 64). The graphite screen printed sensors were commonly constructed using commercially purchased ink consisting of graphite and carbon black particles with a polymeric binder then screen-printed onto a proper substrate and then cured at a proper temperature. These construction steps of the sensor permitted he transfer of electrochemical laboratory experiments to the market for reproducible and disposable on-site determination of different analytes (28, 65). The response of SPE sensor can be easily improved by chemical modification or activation. Based on our knowledge, there are no previous reports for the detection of TM based on SPE or CPE ion selective sensors (ISEs).

Therefore, in this article, new, fast, simple, and cheap potentiometric sensors were made for selective detection of TM in pharmaceutical preparations and biological fluids. This method based on creation and potentiometric characterization of TM-screen-printed and carbon paste sensors using potassium tetrakis ionophore as an electroactive material and *o*-nitro-phenyloctylether (*o*-NPOE) and tricresylphosphate (TCP) as plasticizers. These sensors displayed analytical characteristics with near Nernstian sensitivity and low detection limit. So, proposed sensors could be easily used as indicator sensors in potentiometric titrations of TM in pure form, in tablets, urine and serum samples.

## Experimental

*Reagents and materials*

All chemical and solvents used were of analytical or pharmaceutical grade. Double distilled water was used throughout the experiments. Trimebutine maleate (purity 99.6%) was purchased from ZaCh Systems s.p.a. (Milano, Italy).

Sodium tetraphenylborate (NaTPB) was commercially available from Sigma-Aldrich (USA). Plasticizers used in this study such as dioctylsebacate (DOS), tricresylphosphate (TCP), dibutyl phthalate (DBP),* o-*nitrophenyloctylether (*o-*NPOE), and dioctyl phthalate (DOP) were purchased from Merck (Germany). Relative high molecular weight polyvinyl-chloride (PVC), potassium tetrakis (*p*-chlorophenyl) borate (KTpClPB), and graphite powder (synthetic 1–2 μm) were used for the fabrication of different sensors and they were provided from Aldrich (USA).

*Samples*

Debridate (sample 1; Pfizer, Egypt), Gast-Reg (sample 2; Amoun, Egypt) and Tribudate (Sigma/MPC, Egypt) were used where each tablet contains 200 mg/tablet. Tritone (sample 3; Global Napi, Egypt) was also used where each tablet contains 100 mg/tablet. 

*Apparatus*

Silver-silver chloride Double-Junction reference electrode (Metrohm 6.0726.100) in conjugation with different drug ion-selective electrodes was used. Potentiometric and *p*H-measurements were carried out using 702 titroprocessor equipped with a 665 Dosimat (Switzerland) made by Metrohm. This titroprocessor had a combined electrode, which was more convenient to be used. Digital multimeter connected to a portable PC and Brand digital burette was used for the measurement of the drug under investigation.

*Procedures*

10^−2 ^mol L^-1^ Stock solution of TM drug was prepared by dissolving 0.5035 g of TM in 100 mL bidistilled water. Dilute solutions with the desired concentrations were prepared by accurate dilution from the stock one.

Stock solution of NaTPB (10^-2 ^mol L^-1^) was prepared by dissolving an accurate weighed amount in warm water, adjusted to *p*H 9 by adding sodium hydroxide and completed to the desired volume with water. The resulting solution was standardized potentiometrically against standard thallium(I) nitrate solution (10^-2 ^mol L^-1^). Glucose, lactose, fructose, maltose, starch, sucrose, glycine, urea and chloride salts of Co(II), Ni(II), Ca(II), Mn(II), Zn(II), Na(I), Cd(II), Sr(II), Fe(III), Pb(II), Ba(II), Cu(II) and Al(III) were used as interfering materials and they were purchased from El-Nasr Company, Egypt. 10^−3 ^mol L^−1^ Standard solutions of each of them were prepared in 100 mL bidistilled water.

*Sensors preparation*

*Construction of the modified carbon paste sensors (MCPEs)*

MCPEs were prepared by hand mixing of graphite powder (0.5 g) with 7.5-17.5 mg of potassium tetrakis (*p*-chlorophenyl) borate (KTpClPB) and 5-25 mg multi-wall carbon nanotubes (MWCNTs) and 0.2 mL of different plasticizers (DOP, TCP, DBP, DOS or *o*-NPOE) to a homogenous consistency using a mortar and pestle. The modified carbon paste was packed into the hole of the electrode body and smoothed on a filter paper until it had a shiny appearance and kept in distillated water for 24 h before use (44, 52, 57 and 59).

*Preparation of modified screen printed sensors (MSPEs)*

MSPEs were printed in arrays of six couples consisting of the indicator sensors (each 5 × 35 mm). A polyvinyl chloride flexible sheet (0.2 mm) was used as a substrate which was not affected by the curing temperature or the ink solvent and easily cut by scissors. The indicator sensors were prepared depending on the method of fabrication. The indicator sensor was printed using homemade carbon ink (prepared by mixing 225 mg *o-*NPOE, 0.625 g of polyvinylchloride (8%) and 0.375 g carbon powder). They were printed using homemade carbon ink and cured at 50 °C for 30 min. A layer of an insulator was then placed onto the printed sensors, leaving a defined rectangular shaped (5 × 5 mm) working area and a similar area (for the electrical contact) on the other side. The fabricated sensors were stored at 4 °C and used directly in the potentiometric measurements (26, 45, 51 and 60).

*Potentiometric determination of TM drug in pure solution*

Definite volume of drug solution was pipetted into a 10 mL beaker, 2 mL Britton–Robinson buffer of *p*H 3.0 was added, and the volume was completed to 5 mL with bidistilled water. NaTPB was used as a titrant in the potentiometric titration of the drug under study. The titration process was monitored potentiometrically using the different fabricated sensors. The potential readings were plotted against the volume added of the titrant to estimate the equivalence points. The first derivatives of the titration curves were treated with origin plotting program. The concentration of the drug solutions can be also determined from the constructed calibration curves or using standard addition method.

*Potentiometric determination of TM drug in pharmaceutical samples*

Aliquots of TM solutions were transferred to 10 mL beaker containing 2 mL Britton–Robinson buffer of *p*H 3.0. The content of TM was estimated via potentiometric titration with standard NaTPB solution using the MSPE and MCPE as sensing sensors. The concentration of the drug solutions can be also determined from the constructed calibration curves or using standard addition method.

*Potentiometric determination of TM drug in serum and urine samples*

Tests on urine and serum samples were performed to figure out the amount of urine and serum that give around 100% recovery which determines the effectiveness of this method given that recovery denotes the ratio of the observed value obtained from an analytical process divided by the reference value (26, 27). Phosphate buffer was added to urine or serum samples dropwise until a *p*H 4.0 is obtained. 5 mL of the *p*H-adjusted urine or serum was transferred into six small separatory funnels, and then to each was added 5 mL of 10^−2^, 10^−3^, 10^−4^,10^-5^, 10^-6^ and 10^−7 ^mol L^-1^ standard drug solution, followed by the addition of 20 mL toluene for urine and 20 mL diethyl ether for serum samples, respectively. The urine samples were stored in a refrigerator immediately after their collection. A 10 mL of the sample was centrifuged for 10 min at 2000 rpm. The supernatant was filtered using a 0.45 μm filter and then it was diluted 5-times with phosphate buffer solution of pH 4.0. The solution was transferred into the titration cell to be analyzed without any further pretreatment. The serum samples were obtained and stored frozen until the analysis. In order to precipitate proteins in the plasma samples, 1.0 mL of the samples was treated with 2 mL acetonitrile. Then, the mixture was vortexed for a further 30 s and after that it was centrifuged at 3000 rpm for 10 min. The supernatant was transferred to a small flask and evaporated with the stream of nitrogen. The dry residue was transferred into the titration cell (10 mL) to be analyzed without any further pretreatment. The concentration of the drug can be also determined from the constructed calibration curves or using standard addition method.

## Results and Discussion

*Sensors performance*

**Figure 2:**
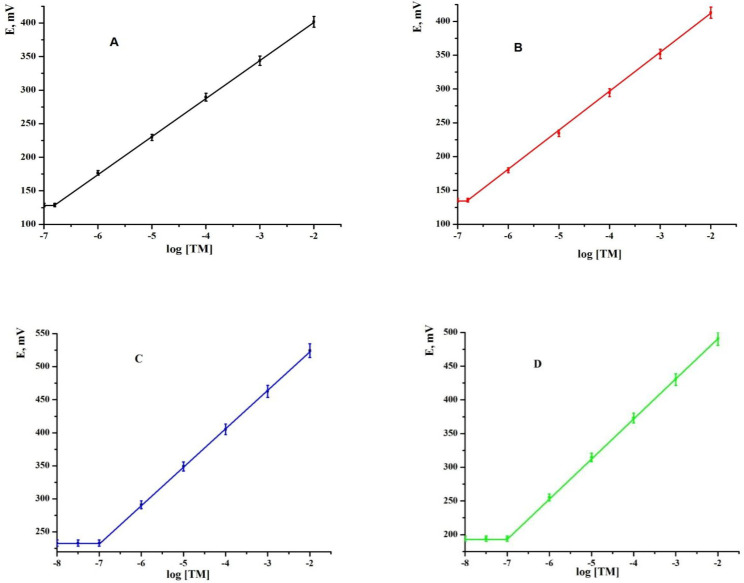
Calibration curve for TM-modified carbon paste sensors [(A) sensor 1 and (B) sensor 2] and for TM-modified screen printed sensors [(C) sensor 3 and (D) sensor 4].

 Several benefit of MCPEs and MSPEs such as they showed very low Ohmic resistance, low cost, very short response time, and reproducibility of the preparation process. Also, they were described by simplicity, cheap and quick preparation process which supports the possibility of measurements on little volumes. This also gave them the benefits for production of a portable titration system. TM-MCPEs (MCPEs; sensors 1 and 2), and TM-MSPEs (MSPEs; sensors 3 and 4) were made using (KTpClPB and MWCNT) as an effective ionophore. Such sensors showed an excellent stability, a broad surface coverage, and a good contact among the prior electrochemical methods. Because of excellent mechanical strength together with supreme heat and electric conductivity of MWCNTs, they have many benefits as high length to diameter ratio, high specific surface area, and high crystallinity. They increased the conductivity of the sensors and decreased the charge transfer between sensor and electrolyte. Appraisal of the potentiometric response properties of both modified CPE and SPE sensors was concluded according to IUPAC recommendations (66). Many experimental parameters such as effect of paste composition, selectivity, working range, pH of the media and life time of sensors were optimized. The response properties of the TM sensors proposed in this study were given in Figure 2. Calibration of the sensors plasticized with TCP at 25 ± 1 °C using the direct calibration technique was carried out. The results proved that the sensors can be successfully implemented for the potentiometric detection of TM in the concentration range from 1.5 × 10^-7^ to 1.0 × 10^-2^ mol L^-1 ^and from 1.0 × 10^-7^ to 1.0 × 10^-2 ^mol L^-1^ for MCPE (tricresylphosphate (TCP, sensor 1), *o*-nitro-phenyloctylether (*o*-NPOE, sensor 2)), MSPE (tricresylphosphate (TCP*, *sensor 3), and* o*-nitrophenyloctylether (*o*-NPOE, sensor 4)) as plasticizers, respectively. They showed Nernstian slope values of 56.77 ± 1.11, 57.82 ± 0.54, 57.95 ± 0.37 and 58.99 ± 0.28 mV decade^-1^ for sensors 1, 2, 3, and 4, respectively. The limit of detection was found to be 1.5 × 10^-7^ and 1.0 × 10^-7 ^mol L^−1^ for MCPEs (sensors 1 and 2) and MSPEs (sensors 3 and 4), respectively.

*Effect of sensor composition*

**Figure 3:**
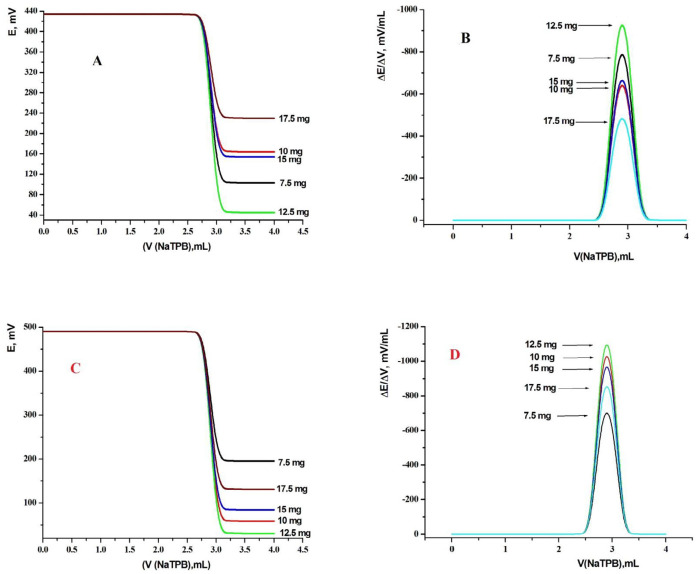
Effect of ionophore content [(A, B) TM-modified Carbon paste sensor and (C, D) TM-modified screen printed Sensors] using TCP plasticizer

Many sensors were made with various compositions. Ionophores or ion exchangers were the most important sensing component in any ion selective electrode. They discerning against interfering ions and selectively bind the target ion. As known the quantity of ionophore in the sensor composition obviously affect the sensitivity and linearity obtained with a given sensor. Also, different ionophore compositions, as shown in Figure 3, were studied. The presence of MWCNTs in composition of the carbon paste improved and increased the conductivity of the sensor, increased transduction of the chemical signal to electrical signal and hence an improvement in the dynamic working range and response time of the sensor will be obtained to Nernstian slope values (26, 27 and 59). On contrary, the sensors without MWCNTs gave a limited

working concentration range, relative high detection limit and showed low sensitivity. So, five modified CPE and SPE sensors containing 7.5, 10, 12.5, 15, and 17.5 mg (KTpClPB and MWCNT) ionophores were synthesized. By running the potentiometric titration for each sensor, the resulting potential breaks at the end point were found to be 327, 266, 385, 276 and 200 mV mL^-1^, and 291, 427, 455, 402 and 355 mV mL^-1^ for MCPEs and MSPEs sensors, respectively. It is clear that the best response in terms of sensitivity and response stability was obtained at the end point (385 and 455 mV mL^-1^) for MCPEs and MSPEs sensors, respectively. This means that with 12.5 mg of (KTpClPB and MWCNT) ionophores, the highest potential break at the end point for MCPEs and MSPEs sensors were obtained. Other electrodes containing more than 12.5 mg of potassium tetrakis (*p*-chlorophenyl) borate (KTpClPB) and MWCNT ionophores were found to have a lower total potential change as shown in Figure 3. 

*Effect of soaking time*

**Figure 4:**
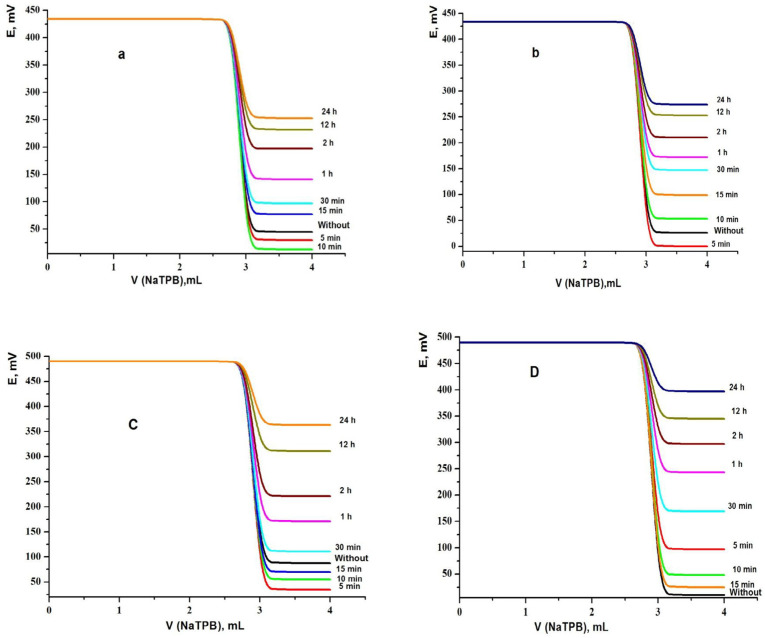
Effect of soaking time on performance of MCPEs [(A) electrode 1 and (B) electrode 2] and MSPEs [(C) electrode 3 and (D) electrode 4]

The performance properties of the fabricated TM-ion-selective sensors were studied as a function of soaking time. Freshly synthesized sensors should be soaked in the produced ion pair to activate their surface. This process required different times based on diffusion at the sensor-test solution interface. A rapid stabilization of equilibrium is certainly a condition for a fast potential response. Therefore, the electrodes 1-4 were soaked in the ion-pair solution of TM, then plot the titration curves from which the total potential changes were recorded after 0 (without soaking), 5, 10, 15, 30 min and 1, 2, 12, 24 h. The data shown in Table 1 pointed out that the best soaking time were 10 and 5 min for sensors 1 and 2, respectively, while the best soaking time was 5 min and without soaking for sensors 3 and 4, respectively. Also, from Table 1 and Figure 4, the potential breaks at the end point were found to be 417, 430, 451, and 476 mV mL^-1^ at 25 ºC for sensors 1, 2, 3 and 4, respectively. It is clear that the total potential change and the potential breaks at the end point for sensors 1-4 decreased with increasing soaking time. So, soaking of these sensors many times is not desirable. This can be ascribed to the lightening of the concentration of the plasticizer and the ionophore in the paste through this contact period. The sensors used in this study must be stored in a refrigerator while not in use. 

*Effect of plasticizer type*

**Figure 5:**
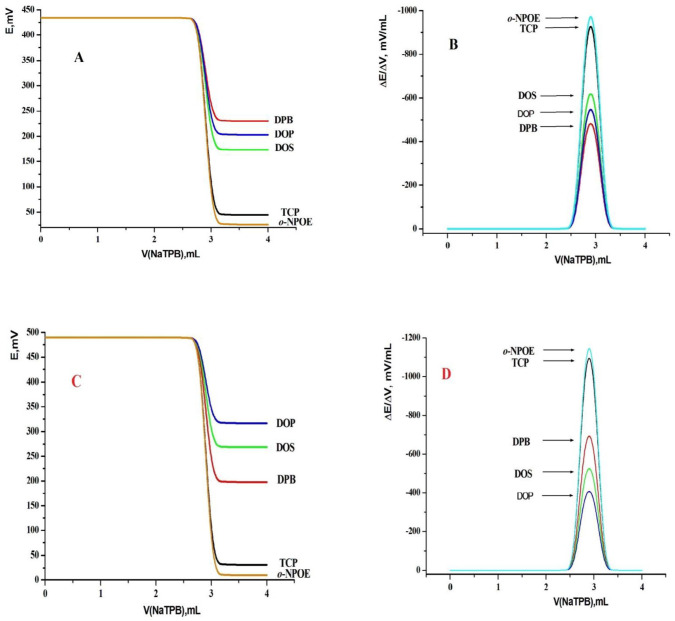
Effect of plasticizer type on the performance characteristics of (A), electrode 1, (B) electrode 2, (C) electrode 3 and (D) electrode 4

The plasticizer content of the printing ink was studied and it must be optimized as it strongly affects the printing process through altering the printing ink viscosity, adhesion, thickness, Ohmic resistance and the sensor analytical performance (26, 27, 44 and 57). The plasticizer content is inversely proportional to the sensor thickness where by increasing the plasticizer content the ink’s viscosity decreased. It is clear that the plasticizer polarity (electric permittivity, ε) affects directly on the selectivity and sensitivity of the MSPE and MCPE, as they decreased the bulk resistance of the electrode because of their polarity characteristics, enhanced the ionic mobility and improved the solubility of the sensing material. By using these sensors as indicator electrodes in the potentiometric titration depending on formation of ion pair, the magnitude of both the potential break and sharpness at the inflexion point of the titration curves can be determined by the plasticizer polarity due to high extractability of the ion pair into the plasticizer (26, 27, 44 and 57). Four standards must be observed for a proper plasticizer such as, selectivity characteristics, sufficient lipophibicity, no crystallization in the paste and no oxidation. Different equilibria between the ionophore and the primary ions in the paste phase could be controlled by the plasticizer, so the performance of sensors can be improved. The nature of the plasticizer could also affect the dielectric constant of the paste and the mobility of the ion-pair. To have the optimum plasticizer for fabricating these sensors, five plasticizers with different dielectric constants called *o*-NPOE, DBP, DOP, DOS, and TCP were used. It is clear from Figure 5 that *o*-NPOE (which has the highest value of dielectric constant) gave the highest total potential change (476 and 404 mV) and the highest potential break at the end point (ΔE/ΔV = 1193 and 1013 mV mL^-1^) for MSPEs and MCPEs, respectively. This may be due to the highest electric permittivity and extractability of the formed NaTPB ion pair into the sensor matrix compared with other tested plasticizers. This led to a short response time which referred to the total time required to reach a stable potential reading through the titration process. No need for a sensor preconditioning before applying the proposed electrodes in the potentiometric titration because of the high extractability of the formed ionophore in the sensor matrix. From the second titration process an excellent titration curves were obtained.

*Response time*

**Figure 6:**
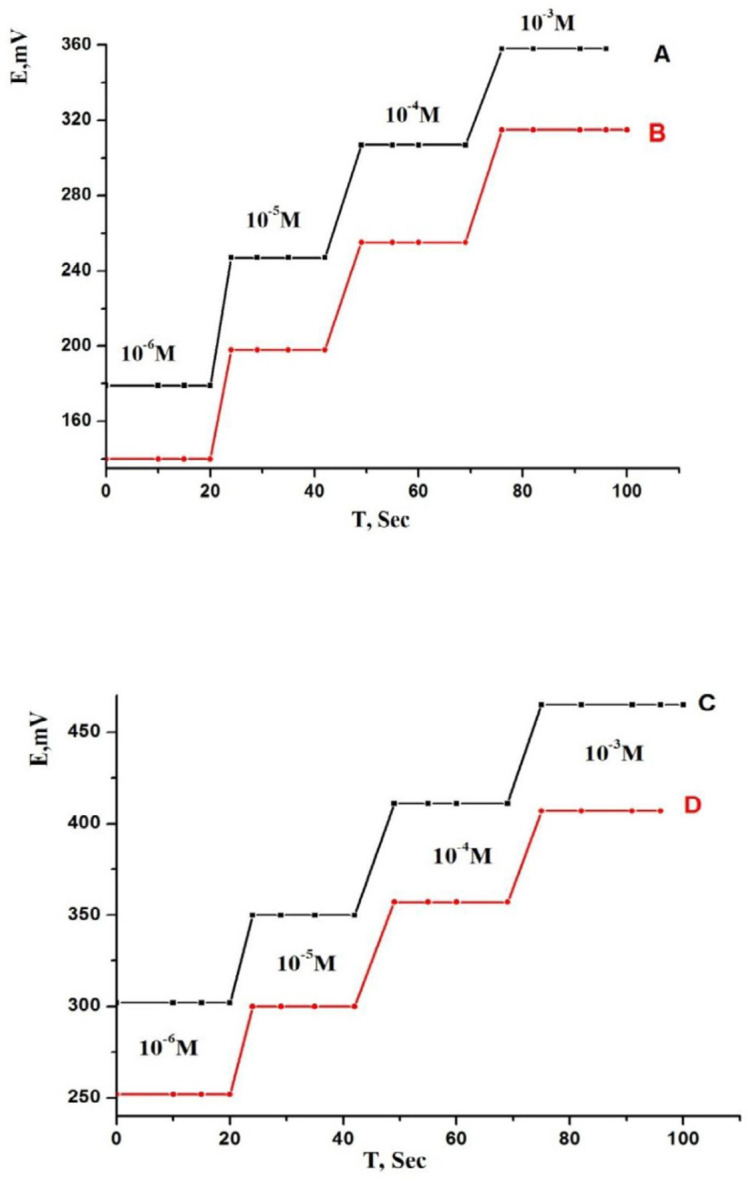
Dynamic response time of (A) electrode 1, (B) electrode 2, (C) electrode 3 and (D) electrode 4

 It is very important parameter as it reported the response properties of a sensor. It is clear that all relevant measurements must be done under the same experimental conditions depending on the membrane type and the interferents. The response time can be considered as the required average time for the sensor to reach a steady potential response within ±1 mV of the final equilibrium value after immersion in a series of TM solutions, each having a 10-fold difference in concentration (37, 67). So, in our study we measured the response time of the sensors by changing the concentration of drug in range from 1.0 × 10^−6^ to 1.0 × 10^−3 ^mol L^-1 ^(Figure 6). From the results, it was found that the optimum response time for the sensors to reach equilibrium was 8 and 6 s for MCPEs and MSPEs, respectively, under the optimized conditions. The presence of MWCNTs in the paste composition led to the fast response of MCPE and MSPE sensors. Therefore, a fast equilibrium was obtained and the sensors will be more hydrophobic to obstruct the formation of water particles on its surface. 

 This led to that the diffusion of the target ions through the sensor surface became very easy (43, 46). The response behavior of the sensors stilled constant when the potentials were recorded either from low to high concentrations or vice versa.

*Life time*

**Figure 7:**
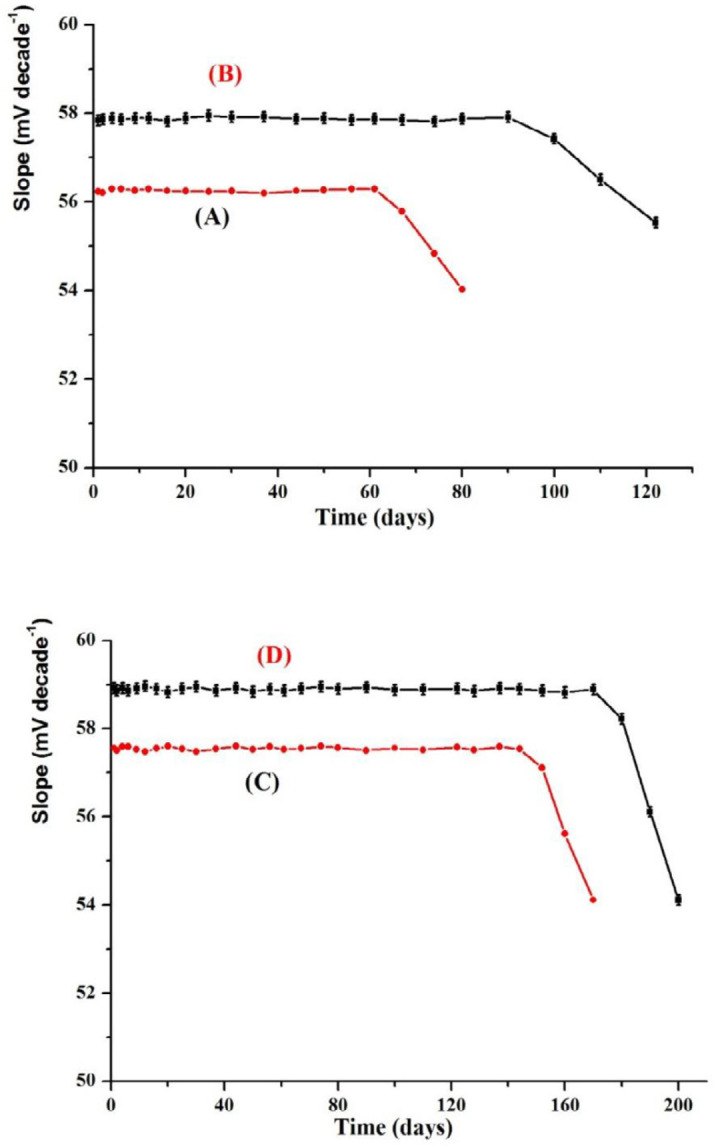
Life time of (A) electrode 1, (B) electrode 2, (C) electrode 3 and (D) electrode 4

 Life time, which is the period in which the electrode functions suitably, can be measured by plotting the calibration curve on different days ranged from one day to 200 days using the proposed electrodes 1-4 (Figure 7). The performance of the fabricated modified SPE and CPE potentiometric sensors can be measured. The results illustrated that there was no measurable deviation or any considerable shift in the value of the slope and these electrodes were allowed to be used after more than 3 and 5 months for MCPEs and MSPEs, respectively (53, 57). This was attributed to the membrane ingredients leakage from the membrane to the solution. This indicated that all the fabricated sensors (I-IV) have high mechanical durability and good adherent to the paste substrate. Each measurement should be run using a new surface and this can be achieved by simply squeezing out a small amount of the paste and polishing the electrode surface on a smooth filter paper till a shiny surface was obtained.

*Effect of pH*

**Figure 8:**
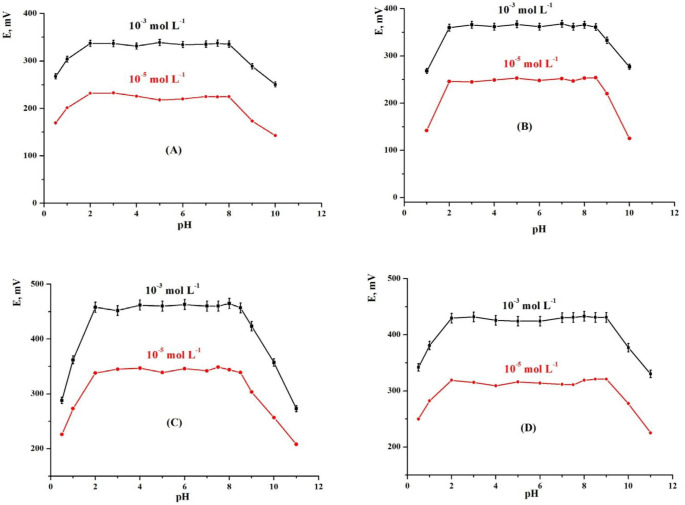
Effect of *p*H of the test solution on (A) electrode 1, (B) electrode 2], (C) electrode 3 and (D) electrode 4

It is an important factor on the current response of the sensor. The effect of *p*H on the potential response of the MCPEs and MSPEs was studied over the *p*H range 1–11 at different concentrations, 1.0 × 10^-3^ and 1.0 × 10^-5 ^mol L^-1^ of TM. The *p*H was adjusted by using HCl or NaOH solution (0.1 – 1 mol L^-1^). It was clear that the electrode potential was independent on pH in the range of 2.0-8.0 and 2.0–8.5 for MCPEs (electrodes 1 and 2) and 2.0-8.5 and 2.0-9.0 for MSPEs (electrodes 3 and 4), respectively (26, 42 and 43). So, this range would be the working pH range for the electrodes 1-4. Acid solutions that have low pH have plenty of hydronium ions that have high mobility and effectively rival with TM drug ions giving high mV readings. While, lower e.m.f. readings were recorded at high *p*H due to the penetration of OH ions and divested the proton from the drug in the paste *i.e.*, depleting charge separation and deteriorating the response. In addition, it may be due to the reaction with the drug ions in the sample (Figure 8). 

*Effect of temperature*

**Figure 9:**
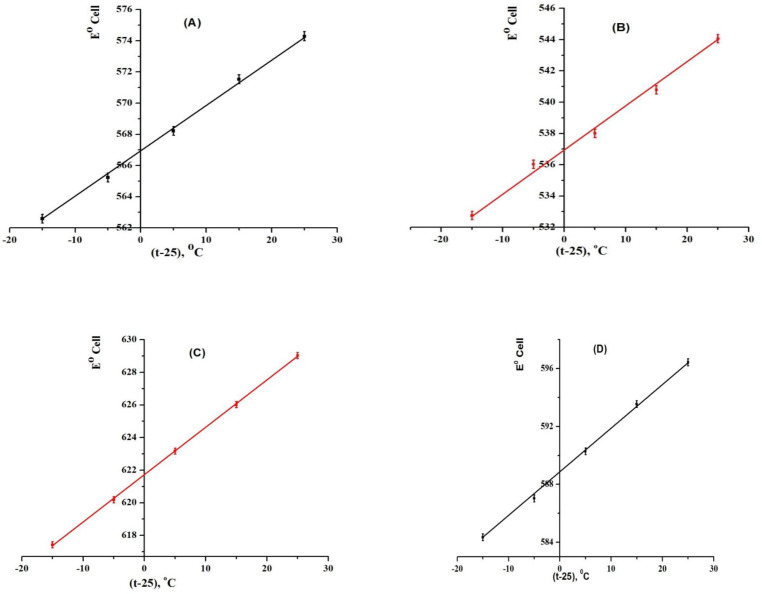
Effect of temperature on the performance of (A) electrode 1, (B) electrode 2], (C) electrode 3 and (D) electrode 4

During characterization of certain sensors (27, 44-46), the effect of temperature and thermal stability must be studied as it may affects the emf response of ion selective sensors and this can be happened by plotting the calibration graphs (E_elect_. versus *p*[TM])) at different test solution temperatures at range 10–50 ºC. From the respective calibration plots the standard cell potentials (E˚_cell_) were obtained at different temperatures as the intercepts of these plots at *p*[TM] = 0 then plotted against (t−25), where t was the temperature of the test solution in ºC giving a straight line plot according to Antropov’s Equation 1 (68) (Figure 9). 

Eº = Eº(25) + (dEº/dt) (t - 25) 

 (Equation 1)

Where E°_(25)_ is the standard electrode potential at 25 °C. The obtained slope of the straight-line acts the isothermal coefficient of MCPE and MSPE electrodes giving values 0.00041, 0.00029, 0.00032, and 0.00021 V/°C for sensors 1, 2, 3 and 4, respectively. This indicated that these electrodes showed a clearly high thermal stability within the investigated temperature range without any observed deviation away from the Nernstian behavior. Temperatures higher than 50 °C caused a significant deviation from the theoretical values and may apparently cause a damage of the sensor surface. Hence, some leaching may be occurred in the paste matrix leading to lowering response of the sensors.

*Selectivity coefficient of the sensors*

 Obviously, the selectivity behavior is one of the most important specifications of the ion-selective electrodes (ISEs) (42, 57 and 58). The selectivity coefficients of the modified SPE and CPE were quantitatively related to equilibria at the interface between the sample and the sensor. Also, they were needed to optimize the paste compositions. The response of the MSPEs and MCPEs to the target drug in the presence of other ions was studied using Nicolsky-Eisenman Equation (2):

${\mathrm{logD}}^{K_{\mathrm{D.B}}^{\mathrm{pot}}=(}E1-E2)/S+(1+z1/z2)\mathrm{log a}$

Where, E_1_ is the potential measured in 1.0 × 10^-3 ^mol L^-1 ^TM (D), E_2 _the potential measured in 1.0 × 10^-3 ^mol L^-1 ^of the interfering compound (B), z_1_ and z_2_ are the charges of the TM (D) and interfering species (B), respectively, S is slope of the electrode calibration plot and *a *is the concentration of the ions used (1 × 10^-3 ^mol L^-1^) and the results obtained were summarized in Table 2. It was clear that the selectivity coefficients largely depend on the composition of the paste and changing widely from one type to another. K_B,D _may have values varying from zero (no interference) to greater than unity (the sensor responds to the interferent more than to the primary species). The potential measured by a potentiometric sensor was mostly affected by the primary ion but a contribution from other ions which can interact with the sensor may be occurred. This contribution would be very small or negligible. Both separate solutions and matched potential methods (SSM and MPM) were studied in this study to specify the selectivity coefficient of the sensors towards the target drug in the presence of various interferents (42, 57 and 58) using 1.0 × 10^-3 ^mol L^-1 ^concentration for both of the standard drug and the interference. In SSM, the Nicolsky coefficient could be specified by comparing potential of two solutions, containing the primary and interfering ion only, the selectivity coefficient is determined using the Nicolsky-Eisenman equation. While in MPM (36), the potentiometric selectivity coefficient was defined as the activity ratio of primary (TM) and interfering (B) ions that gave the same potential change under identical conditions. 

The selectivity coefficient could be determined with the following Equation 3: 

$K_{\mathrm{TM.B}}^{\mathrm{MPM}}=(C_{\mathrm{TM}}-C_{{\mathrm{TM}}^{+}})/C_{B}$

(Equation 3)

Where C_B_ is the concentration of the interfering ion, C_TM_ is the initial primary ion concentration (1.0 × 10^-3 ^mol L^-1^) and C`_TM_ is the new concentration of TM after addition of an aliquot of interfering ions. A sensible selectivity toward TM in the presence of many carbohydrates and nitrogenous compounds such as amines, glycine, and some inorganic cations was noted. The results showed no diligent interference by a number of pharmaceutical excipients, diluents and active ingredients commonly used in the drug formulations (*e.g.* glucose, lactose, maltose, fructose, starch and sucrose) at concentration as high as a 10–100-fold molar excess over TM. The results shown in indicated that the constructed sensors displayed high selectivity for TM over common drugs and most of the selectivity coefficients were very low, indicating no significant interference in the performance of the modified electrodes for determination of TM drug (Table 2).

*Analytical applications to drug analysis*

To illustrate the fitting of the developed MCPE and MSPE plasticized with TCP and *o*-NPOE for routine analysis; the sensors 1-4 were applied to specify TM drug concentration in pharmaceutical formulations, such as Debridate, Gast-Reg, Tritone, and Tribudate tablets using the calibration curve and potentiometric titration methods. The results were summarized in Table 3 and compared to the British Pharmacopeia (26, 27). It indicated that there was no significant difference between the proposed and reported method in terms of F- and *t*-test values Table 3. The percentage of recovery values, relative standard deviation and standard deviation were listed in Table 3 and the low values of standard deviation and relative standard deviation in comparison with the official method indicated the high accuracy and precision of the proposed potentiometric method. 

*Application to serum and urine*

Determination of TM in biological fluids such as human serum and urine was carried out using the proposed method in order to check the capability of the proposed sensors for the determination of TM in the spiked samples which were prepared from serial concentrations of TM reference standards. Four measurements were performed for each concentration as seen in Table 4. It was clear that good recovery values and low RSD were obtained indicating that the proposed sensors in this work were suitable for TM determination in serum and urine samples with high sensitivity, precision and without interferences from the co-formulated adjuvants as indicated by the percentage recovery values.

*Method validation*

 The method was validated for accuracy, precision, repeatability, robustness, and ruggedness in accordance with ICH guidelines (52, 58).

*Accuracy*

It is the closeness between the true or accepted reference value and the obtained value (69). The accuracy of the proposed method utilizing MSPEs and MCPEs sensors (sensors 1-4) was investigated by specifying of TM in spiked samples prepared from serial concentrations of TM reference standards. The results were summarized in Table 5. The high percentage recovery values indicated the high accuracy of the proposed method for the identification of TM drug in its sample’s preparations without interferences from the co-formulated adjuvant.

*Precision*

It is the degree of repeatability of an analytical method and used as a measure of how close results are to one another. It could be defined as the closeness of agreement between independent test results obtained under stipulated conditions. Precision was usually expressed as standard or relative standard deviations of the replicate analysis (69). Hence, percentage relative standard deviation (RSD%) was used to measure the precision of the proposed potentiometric method using the sensors under investigation. Three concentrations and five replicates of each concentration were used for intra-day (on the same day) and inter-day (on different days) precisions assessment. Low values of relative standard deviations and standard deviation reflected to reasonable reproducibility, precision, repeatability, and the good accuracy of the proposed method (Table 5). 

*Robustness/ruggedness of the sensor*

 It was obvious that MCPE and MSPE were stable towards mechanical shocks with increasing the temperature up to 50 ºC also; they have a great benefit of the renewal of its surface without changing its properties. 

**Table 1:** Effect of soak1ng t1me on the performance of MCPEs [electrodes 1 and 2] and MSPEs [electrodes 3 and V1].

**Electrode type**	**T1me of soak1ng**	**End po1nt (mL)**		**Recovery (%)**		**Potent1al break** **at the end po1nt, (mV)**		**ΔE/ΔV** **(mV /mL)** ^-1^	
		**Sensor 1**	**Sensor 2**	**Sensor 1**	**Sensor 2**	**Sensor 1**	**Sensor 2**	**Sensor 1**	**Sensor 2**
MCPEs	W1thout	2.983	2.994	99.43	99.80	385	404	965	1013
	5 m1n	2.992	2.998	99.73	99.93	400	430	1003	1063
	10 m1n	2.995	2.986	99.83	99.53	417	377	1045	945
	15 m1n	2.948	2.963	98.26	98.76	353	331	885	830
	30 m1n	2.922	2.939	97.40	97.96	333	283	835	710
	1 h	2.883	2.904	96.10	96.80	289	258	725	648
	2 h	2.847	2.876	94.90	95.86	234	220	588	553
	12 h	2.807	2.835	93.56	94.50	198	177	498	445
	24 h	2.767	2.799	92.23	93.30	177	156	445	393
		**Sensor 3**	**Sensor 4**	**Sensor 3**	**Sensor 4**	**Sensor 3**	**Sensor 4**	**Sensor 3**	**Sensor 4**
MCPEs	W1thout	2.986	2.999	99.53	99.97	398	476	998	1193
	5 m1n	2.998	2.965	99.93	98.83	451	389	1130	975
	10 m1n	2.996	2.989	99.87	99.63	431	438	1080	1098
	15 m1n	2.991	2.992	99.70	99.73	416	461	1043	1155
	30 m1n	2.949	2.953	98.30	98.43	375	317	940	795
	1 h	2.923	2.937	97.43	97.90	315	243	790	610
	2 h	2.882	2.898	96.06	96.60	265	189	665	475
	12 h	2.855	2.861	95.16	95.36	175	141	440	355
	24 h	2.803	2.829	93.43	94.30	123	89	310	225

**Table 2:** Potentiometric select4ity coefficients of some interfering ions using MCPE [1 and 2] and MSPEs [3 and 4] Sensors

**Interfering ions**	${-\mathrm{log k}}_{{\mathrm{TM}}^{+}\mathrm{. B}}^{\mathrm{MPM}}$				${-\mathrm{log k}}_{{\mathrm{TM}}^{+}\mathrm{. B}}^{\mathrm{SSM}}$			
	**MCPEs**		**MSPEs**		**MCPEs**		**MSPEs**	
	**Sensor 1**	**Sensor 2**	**Sensor ** **3**	**Sensor** **4**	**Sensor 1**	**Sensor 2**	**Sensor 3**	**Sensor 4**
Maltose	4.32	4.78	4.72	4.81	3.95	4.23	4.21	4.40
Lactose	4.41	4.89	4.89	5.01	4.32	4.54	4.39	4.98
Glucose	4.47	4.54	4.41	4.63	4.27	4.44	4.32	4.51
Sucrose	4.56	4.97	4.81	4.99	4.43	4.61	4.72	4.87
Glycine	4.79	5.24	5.07	5.31	4.55	4.69	4.86	4.93
Fructose	5.04	5.27	5.23	4.72	4.78	4.98	5.11	5.43
Starch	3.59	3.86	3.99	4.23	3.65	3.76	3.91	4.02
Urea	3.61	3.88	3.85	3.99	3.48	3.59	3.62	3.98
Al^3+^	3.57	3.79	3.98	3.89	3.42	3.73	3.88	4.12
Fe^3+^	3.63	3.75	3.86	3.77	3.55	3.67	3.77	3.92
Ca^2+^	4.63	4.67	4.73	4.81	4.45	4.53	4.62	4.88
Cu^2+^	3.40	3.74	3.52	3.94	3.22	3.47	3.51	3.71
Sr^2+^	4.61	4.64	4.69	4.81	4.33	4.41	4.52	4.59
Mn^2+^	3.99	4.17	4.25	4.32	3.75	3.89	4.02	4.11
Ba^2+^	4.27	4.43	4.51	4.54	4.12	4.34	4.44	4.51
Co^2+^	4.56	4.64	4.83	4.71	4.48	4.51	4.67	4.72
Cd^2+^	4.32	4.66	4.55	4.78	4.12	4.31	4.52	4.63
Pb^2+^	4.93	5.28	5.27	5.31	4.73	5.01	5.22	5.31
Ni^2+^	4.57	4.96	4.69	4.82	4.37	4.43	4.56	4.59
Na^+^	4.71	4.99	4.91	4.45	4.55	4.68	4.76	4.88
Zn^2+^	4.59	5.13	5.27	3.81	4.64	4.88	5.01	5.12

**Table 3:** Potentiometric determination of TM in pharmaceutical formulations using MCPEs [electrodes 1 and 2] and MSPEs [electrodes 3 and 4].

MCPEs	**Samples** ^b^	**[TM] Taken** **(mg mL** ^-1^ **)**	**Values**									**British Pharmacopiea**				
			**Sensor 1**			**Sensor 2**										
			**Found** **(mg mL** ^-1^ **)**	**Recovery** ** (%)**	**RSD** ^a^ **(%)**	**Found** **(mg mL** ^-1^ **)**		**Recovery (%)**		**RSD** ^a^ **(%)**		**Found** **(mg mL** ^-1^ **)**	**Recovery** ** (%)**		**RSD** ^a^ **(%)**	
	(1)	2.25	2.20	97.77	1.29	2.22		98.67		1.11		2.20	97.78		1.86	
	(2)	2.35	2.34	99.57	0.978	2.36		100.4		0.72		2.29	97.45		2.17	
	(3)	2.25	2.22	98.66	1.09	2.23		99.11		0.88		2.21	98.22		1.73	
	(4)	2.30	2.27	98.69	1.03	2.26		98.26		0.71		2.24	97.39		1.71	
	SD		0.049 - 0.351			0.034 - 0.205						0.082-0.629				
	F-test^#^		0.99-2.29			0.51-1.87										
	*t*-test^#^		1.64-2.78			1.07-2.41										
			**Sensor 3**			**Sensor 4**										
			**Found** **(mg mL** ^-1^ **)**	**Recovery** ** (%)**	**RSD** ^a^ **(%)**	**Found** **(mg mL** ^-1^ **)**		**Recovery** ** (%)**		**RSD** ^a^ **(%)**		**Found** **(mg mL** ^-1^ **)**	**Recovery** ** (%)**		**RSD** ^a^ ** (%)**	
MSPEs	(1)	2.25	2.23	99.11	1.0		2.24		99.56			2.21		98.22		1.32
	(2)	2.35	2.35	100.00	0.43		2.35		100.0			2.30		97.87		1.86
	(3)	2.25	2.24	99.56	1.01		2.23		99.11			2.22		98.67		1.42
	(4)	2.30	2.28	99.13	0.99		2.29		99.57			2.26		98.26		1.44
	SD		0.009–0.127				0.003–0.34					0.041–0.211				
	F-test^#^		0.48–1.54				0.31–0.95									
	*t*-test^*^		0.72–1.81				0.41–1.11									

^a^number of replicates is 4.

^#^Tabulated F value at 95% confidence limit = 5.984 for n = 4 for MCPEs and 5.267 for n = 4 for MSPEs.

^*^Tabulated *t* value at 95% confidence limit = 2.44 for n = 4 for MCPEs and 2.35 for n = 4 for MSPEs.

^b^Samples (1-4) are described in details in the experimental part.

**Table 4:** Determination of TM in spiked urine and human serum using MCPEs and MSPEs

	**Sample**	**Statistical** **parameters**	**(Sensor 1)**			**(Sensor 2)**		
MCPEs	Urine		**Direct** **Method**	**Calibration** **Graphs**	**Standard addition** **method**	**Direct** **method**	**Calibration graphs**	**Standard addition** **method**
		Mean recovery (%)	98.37	98.68	98.41	99.11	99.26	98.81
		N	4	4	4	4	4	4
		Variance	0.83	0.86	0.85	0.92	0.83	0.98
		RSD (%)	1.12	1.01	1.43	1.33	0.94	1.66
	Serum	Mean recovery (%)	98.55	98.72	98.20	99.23	99.36	99.0
		N	4	4	4	4	4	4
		Variance	0.64	0.58	0.61	0.71	0.49	0.69
		RSD (%)	1.02	0.86	1.37	1.24	0.68	1.86
MSPEs	Urine		**(Sensor 3)**			**(Sensor 4)**		
			**Direct** **Method**	**Calibration** **Graphs**	**Standard addition** **method**	**Direct** **method**	**Calibration graphs**	**Standard addition** **method**
		Mean recovery (%)	99.21	99.44	98.89	99.87	99.77	99.11
		N	4	4	4	4	4	4
		Variance	0.91	0.88	0.91	0.97	0.92	0.88
		RSD (%)	1.01	0.81	1.22	1.05	0.63	1.36
	Serum	Mean recovery (%)	99.01	99.57	99.21	99.88	99.89	99.25
		N	4	4	4	4	4	4
		Variance	0.77	0.66	0.74	0.86	0.51	0.67
		RSD (%)	0.87	0.68	1.07	0.88	0.38	1.01

**Table 5.** Evaluation of intra- and inter-days precision and accuracy of MCPEs and MSPEs

**Sensor type**	**Sample No.**	**[TM] Taken, (mg/mL)**	**Intra day [TM] Found, (mg/mL)**	**Recovery (%)**	**SD**	**RSD (%)**	**Inter day [TM] Found, (mg/mL)**	**Recovery (%)**	**SD**	**RSD (%) **
MCPE (Electrode 2)	Pure [TM]	0.35	0.350	100.0	0.002	0.795	0.349	99.71	0.008	1.321
		0.50	0.497	99.40	0.021	1.012	0.496	99.20	0.038	1.412
	Sample2	0.35	0.346	98.86	0.028	1.124	0.345	98.57	0.055	1.641
		0.50	0.498	99.60	0.015	1.512	0.495	99.00	0.042	1.952
	Sample 4	0.35	0.347	99.14	0.036	1.345	0.347	99.14	0.061	1.857
		0.50	0.499	99.80	0.012	1.311	0.495	99.00	0.021	1.444
MSPE (Electrode 4)	Pure [TM]	0.35	0.351	100.3	0.001	0.652	0.349	99.71	0.069	1.012
		0.50	0.50	100.0	0.006	0.956	0.5	100.0	0.124	0.779
	Sample2	0.35	0.349	99.71	0.152	1.186	0.349	99.71	0.235	0.998
		0.50	0.498	99.60	0.665	1.687	0.497	99.40	0.558	1.214
	Sample 4	0.35	0.349	99.71	0.235	1.952	0.351	100.3	0.687	1.698
		0.50	0.498	99.60	0.102	0.645	0.498	99.60	0.214	1.001

**Table 6:** Response characteristics of MCPEs (electrodes 1 and 2) and MSPEs (electrodes 3 and 4).

**Parameter**	**MCPEs**		**MSPEs**	
	**Electrode 1**	**Electrode 2**	**Electrode 3**	**Electrode 4**
Slope (mV decade^-1^)	56.77 ± 1.11	57.82 ± 0.73	58.02 ± 0.66	58.91 ± 0.21
Concentration range (mol L^-1^)	1.5 × 10^-7^ to 1.0 × 10^-2^	1.5 × 10^-7^ to 1.0 × 10^-2^	1.0 × 10^-7^ to 1.0 × 10^-2^	10 × 10^-7^ to 1.0 × 10^-2^
Correlation coefficient, r	0.99978	0.99989	0.99997	0.99999
Limit of detection (mol L^-1^)	1.5 × 10^-7^	1.5 × 10^-7^	1.0 × 10-7	1.0 × 10^-7^
Limit of quantification (mol L^-1^)	4.95 × 10^-7^	4.95 × 10^-7^	3.3 × 10^-7^	3.3 × 10^-7^
Working *p*H range	2.0 – 8.0	2.0 – 8.5	2.0 – 8.5	2.0 – 9.0
Response time (sec)	9	8	7	6
Life time (months)	2	3	5	6
Standard deviation (SD)	0.678 – 0.986	0.365- 0.656	0.098- 0.404	0.067- 0.231
Relat4e standard deviation (RSD %)	1.124 – 1.779	1.013 – 1.227	1.001- 1.077	0.879 – 1.002
Intercept (mV)	514.89 ± 1.85	526.10 ± 2.59	638.34 ± 1.78	608.59 ± 1.35

## Conclusion

In this paper a convenient simple, sensitive and rapid potentiometric method was developed for estimation of TM either in urine, serum or in pharmaceutical dosage forms by preparation of modified screen printed and carbon paste sensors. The proposed sensors have good characteristics, such as near-Nernstian response over a wide concentration range (1.5 × 10^-7^ - 1.0 × 10^-2 ^and 1.0 × 10^-7^- 1.0 × 10^-2^ mol L^-1^) at 25 °C with low detection limit (1.5 × 10^-7^ and 1.0 × 10^-7 ^mol L^-1 ^for MCPEs and MSPEs, respectively) (Table 6). The fabricated sensors exhibited high sensitivity, reasonable selectivity, fast static response, high stability and could be applied over a wide *p*H range with minimal sample pretreatment. Also, a good selectivity for TM with respect to some inorganic cations, sugars and glycine was obtained. The used method in this paper ensured the high accuracy to assay TM in tablets, urine and serum samples. The accuracy of this method was indicated by excellent recovery and low standard deviation values.
